# BRCA2 Deletion Induces Alternative Lengthening of Telomeres in Telomerase Positive Colon Cancer Cells

**DOI:** 10.3390/genes10090697

**Published:** 2019-09-10

**Authors:** Luca Pompili, Carmen Maresca, Angela Dello Stritto, Annamaria Biroccio, Erica Salvati

**Affiliations:** 1 Oncogenomic and Epigenetic Unit, IRCSS Regina Elena National Cancer Institute, Via Elio Chianesi, 53-00144 Rome, Italy; luca.pompili@ifo.gov.it (P.L.); Carmen.maresca@ifo.gov.it (M.C.); Annamaria.biroccio@ifo.gov.it (B.A.); 2 Biology and Biotechnology Department “Charles Darwin”, Sapienza University of Rome, Piazzale Aldo Moro, 5-00185 Rome, Italy; angela.dellostritto@uniroma1.it; 3 Institute of Molecular Biology and Pathology, CNR, Via degli Apuli, 4-00185 Rome, Italy

**Keywords:** BRCA2 mutation, telomeres, ALT

## Abstract

BRCA1/2 are tumor suppressor genes controlling genomic stability also at telomeric and subtelomeric loci. Their mutation confers a predisposition to different human cancers but also sensitivity to antitumor drugs including poly(ADP-ribose) polymerase (PARP) inhibitors and G-quadruplex stabilizers. Here we demonstrate that BRCA2 deletion triggers TERRA hyperexpression and alternative lengthening mechanisms (ALT) in colon cancer cells in presence of telomerase activity. This finding opens the question if cancer patients bearing BRCA2 germline or sporadic mutation are suitable for anti-telomerase therapies, or how ALT activation could influence the short or long-term response to anti-PARP inhibitors or anti-G-quadruplex therapies.

## 1. Introduction

Telomeres are nucleoprotein structures at the end of eukaryotic chromosomes preserving genome integrity [1]. They consist of species-specific CG-rich repeats, (TTAGGG)n in humans, and exhibit heterochromatic marks [2,3]. Telomeric repeats are bound by a six-member protein complex called shelterin that masks the end of linear DNA molecules avoiding detrimental recombination events and chromosomal instability [4]. Telomeres lose DNA repeats at each duplication round until they reach a critical length driving replicative senescence in normal cells or apoptosis in tumor cells [5,6,7]. To overcome telomere erosion, cancer cells reactivate telomere maintenance mechanisms (TMM), which in the majority of cases consist of reactivation of the telomerase holoenzyme, a retrotranscriptase which add repeats to DNA ends by using an RNA template [8]. In a certain number of human cancers, transformed cells adopt telomere-specific homologous recombination (HR) using the sister chromatid as a template for telomere elongation [9]. More than one molecular mechanisms have been ascribed to these telomerase-independent TMM which are collectively called ALT [10]. ALT positivity of cancer cells is more difficult to be assessed than telomerase positivity since a series of different parameters have to be considered: high and heterogeneous telomeres length, presence of ALT-associated PML bodies (APBs), and presence of DNA circles containing telomeric repeats as refuse of homologous recombination [11]. In addition, telomerase and ALT mechanisms have been shown to coexist in cancer cells [12,13]. It has been hypothesized that epigenetic background of cells can influence their commitment to reactivate telomerase or to trigger ALT, since tumors with mesenchymal origin are more frequently ALT, whereas epithelial ones are more frequently telomerase positive [14]. ALT positivity is also associated with alteration of expression of chromatin remodeling factors with subtelomeric localization as the ATRX/DAXX/H3.3 complex [15]. In addition, the demethylated state of subtelomeric regions in ALT cells results in a de-repression of the telomere-specific lncRNAs (TERRA) promoters, which accumulates at very high levels [16]. In this regard, it has been proposed that TERRA expression could play a role in increasing the recombination frequency of ALT telomeres, contributing to maintain their length [17]. 

HR is an extremely conserved pathway evolved for preserving genome integrity in case of DNA double-strand breaks (DSBs) induced by exogenous or endogenous insult. Unrepaired damage leads to deletions, frameshifts, chromosome aberrations, and aneuploidy [18]. DSB is first recognized by poly(ADP-ribose) polymerase 1 (PARP1), that marks the damage site by attaching ADP-ribose molecules to chromatin-bound proteins surrounding the break [19]. The ADP-ribose units are essential for recruitment of the MRN complex (consisting of Mre11, Rad50 and Nbs1 proteins) that mediates resection and allows recruitment of the single-strand binding protein replication protein A (RPA). RPA stabilizes the newly produced ssDNA overhangs which allow the recruitment of other factors (BRCA1/2, PALB2, and RAD51 paralogs) which in turn mediate strand invasion into a homologous DNA region, usually a sister chromatid, enabling repair to be completed [20]. One of the crucial proteins involved in this process is the breast cancer type 2 susceptibility factors BRCA2, which is responsible for RAD51 loading on the ssDNA overhang. Germline mutation of BRCA2 (as well as BRCA1) is responsible for the hereditary breast and ovarian cancer (HBOC) syndrome which is a highly penetrant autosomal dominant disorder accounting for 5–7% of breast cancers (BCs) and 8–13% of epithelial ovarian cancers (EOCs) [21]. The detection of genomic rearrangements by whole-genome sequencing in BRCA1/BRCA2-deficient samples lead to identification of six distinct mutational signatures that correlated with BRCA status [22]. Notably, the so-called BRCA-ness signature was identified in different type of cancers that did not have detectable BRCA1/BRCA2 mutations, connecting genomic rearrangements with functional HR deficiency, and suggesting that additional molecular alterations might underline BRCA-like phenotypes in several cancer histotypes [22].

In addition to genomic rearrangements, telomere length is a measurement of genome instability [23]. HR repair proteins function to protect telomere regions from damage [24], and telomere defects are often observed in genome unstable cells [25]. BRCA2 was shown to load RAD51 during telomeric replication and its absence induces telomere shortening and breakage [26] due to activation of improper recombination. BRCA2 is in fact considered as a suppressor of HR at telomeres in telomerase positive cells. In ALT cells, instead, BRCA2 loss of function could favor telomere length maintenance through recombination [27]. Here we will address the point if BRCA2 deletion could affect ALT dependent telomere maintenance in an isogenic cell model of colon cancer.

## 2. Materials and Methods

### 2.1. Cell Culture

BRCA2 deficient and proficient DLD1 (*BRCA2*Δ*ex11/*Δ*ex11*, Horizon Discovery [28]) and shSCR/shBRCA2 stably transfected HCT colon cancer cell lines were a kind gift of Dr. Madalena Tarsounas [29]. U2OS and SaOS osteosarcoma, HCT116 colorectal carcinoma cells were purchased from ATCC repository. Cells were maintained in Dulbecco Modified Eagle Medium (D-MEM, Invitrogen Carlsbad, CA, USA) supplemented with 10% fetal calf serum, 2 mM L-glutamine, and antibiotics. 

### 2.2. Real-Time Quantitative-Telomerase Repeat Amplification Protocol Assay (RTQ-TRAP)

The SYBR Green RQ-TRAP assay was conducted as described in Reference [30] with 0.5 μL of cell extract (1000 cells/μL). Primer sequences were described by Kim and Wu [31]. Using the 7900HT Fast Real-Time PCR System (Applied Biosystem, Waltham, MA, USA), samples were incubated for 30 min at 30 °C and amplified in 40 PCR cycles with 30 s at 95 °C and 90 s at 60 °C (two-step PCR). The threshold cycle values (Ct) were determined from semi-log amplification plots (log increase in fluorescence vs cycle number) and compared with standard curves generated from serial dilutions of HCT116 cell extracts (1000, 500, 250, 50 cells). Each sample was analyzed in triplicate. Telomerase activity was expressed as fold changes of HCT116 500 cells value (Relative Telomerase Activity).

### 2.3. TERRA Northern Blot

RNA (15 μg/sample) was electrophoresed on an agarose gel and transferred onto a nylon membrane for northern blotting analysis. Briefly, RNA (15 μg/sample) was denatured in 2.3× denaturant (2.5 mL 40× MOPS, 2.5 mL H_2_O, 35 mL formaldehyde, 100 mL deionized formamide) for 15 min at 65 °C and separated by 1.2% agarose formamide gel in 1× MOPS buffer at 120 V. Electrophoresis was stopped when bromophenol blue dye reached 8 cm from wells. After electrophoresis, RNA samples were transferred on nylon positively charged membrane (GE Healthcare UK Limited, Little Chalfont, UK) with 20× SSC overnight and UV cross-linked onto membrane at 125 mJ in UV crosslinker (Hoefer, Holliston, MA, USA). For RNA detection, the blot was hybridized with a 32P-labeled probe [T3AG2] in Church buffer (0.5 M phosphate buffer pH 7.2, 7% SDS, 1 mM EDTA, 0.1% BSA) overnight at 55 °C. The gel was washed twice and exposed to a PhosphorImager screen and analyzed by Quantity One software (Biorad, Hercules, CA, USA).

### 2.4. C-Circle Assay

Total DNAs were extracted using Quick C-Circle Lysis Buffer (50 mM KCl, 10 mM Tris HCl pH 8.5, 2 mM MgCl_2_, 0.5% NP40, 0.5% Tween 20) and treated with 0.5 µg µL^−1^ protease (Qiagen, Hilden, Germany). Samples, 10 μL each, were combined with 10 μL 0.2 mg/mL BSA, 10% Tween, 100 mM each dATP, dGTP, and dTTP, 1 × Φ29 Buffer and with or without 7.5 U Φ29 DNA polymerase (NEB) and incubated at 30 °C for 4 h then 70 °C for 20 min. For quantification, the reaction products were dot-blotted onto a 2× SSC-soaked nylon positively charged membrane (GE Healthcare UK Limited, Little Chalfont, UK). DNA was UV-cross-linked onto the membrane, which was then hybridized with a 32P-labeled probe [T3AG2] in Church buffer (0.5 M Phosphate buffer pH 7.2, 7% SDS, 1 mM EDTA, 0.1% BSA) overnight at 55 °C. The gel was washed twice and exposed to a PhosphorImager screen and analyzed by Quantity One software (Biorad, Hercules, CA, USA).

Signals of samples without Φ29 DNA polymerase were subtracted from signal obtained from corresponding samples with Φ29 DNA polymerase to determine the DNA c-circles expression value.

### 2.5. Real-Time qPCR

Real-time qPCR analysis of TERRA was performed as described [32]. Briefly, RNA was extracted from cells with RNAeasy mini kit (Qiagen) and accurately digested with the RNase-free DNase set (Qiagen). Then, RNA quality was checked on FA gel electrophoresis and amplified in real-time PCR assay with subtelomere-specific primers with a 7900 HT Fast Real-Time PCR System (Applied Biosystem, Waltham, MA, USA). 

### 2.6. IF-FISH

Cells were fixed in 2% formaldehyde and permeabilized in 0.25% Triton X-100 in PBS for 5 min at room temperature. For immunolabeling, cells were incubated with anti-PML (goat polyclonal N19 Santa Cruz Biotechnology) and anti-TRF1 (rabbit polyclonal N19, Santa Cruz Biotechnology, Dallas, TX, USA) primary antibodies at room temperature for 2 h, then washed in PBS and incubated with the FITC-conjugated bovine anti-goat (Santa Cruz Biotechnology, Dallas, TX, USA) followed by cy3-conjugated donkey anti-rabbit secondary antibodies (Jackson Immunoresearch). For FISH analysis, cells fixed as above were dehydrated and hybridized with Cy3-coupled PNA telo probe (Panagene) as described by Lenain et al. (2006). Images were captured with an X100 objective. Fluorescent signals were recorded by using a Leica DMIRE2 microscope equipped with a Leica DFC 350FX camera and elaborated by a Leica FW4000 deconvolution software (Leica, Solms, Germany). This system permits to focus single planes inside the cell, generating 3D high-resolution images. For qFISH analysis, telomeric spots intensity was calculated by ImageJ software on images acquired at the same exposure time. 

## 3. Results

### 3.1. BRCA2 Depletion Induces TERRA Transcription

**Figure 1:**
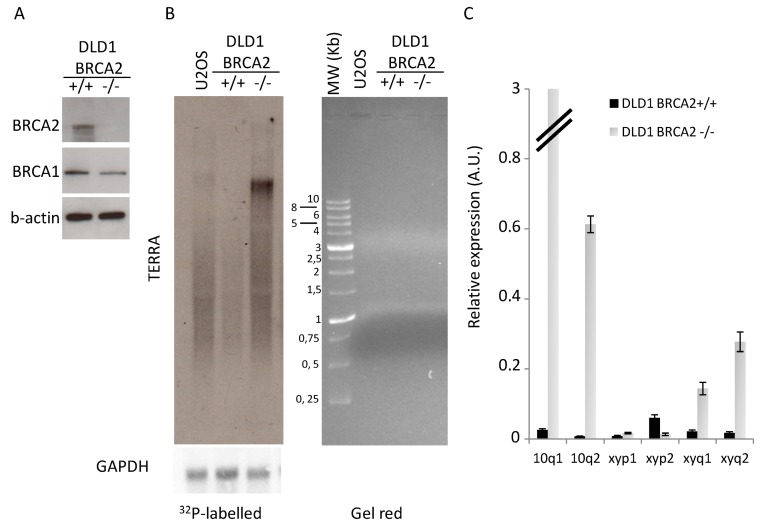
TERRA expression analysis. (**A**): Western blot analysis of BRCA2 expression levels in proficient and deficient DLD1 b-actin is shown as loading control. One representative of three independent experiments is shown (**B**): Northern blot analysis of TERRA expression (GAPDH is shown as loading control), RNA separation on agarose gel is shown on the left panel. One representative of three independent experiments is shown. (**C**): qPCR analysis of TERRA expression at different subtelomeres. The mean of three independent experiments with comparable results is shown. Bars are SD.

Telomere homeostasis is affected by BRCA1/2 mutations as a consequence of global increase of genomic instability, due to alteration of DNA repair pathways, and for direct implication of BRCA2 in telomere replication [24,25,26]. Moreover, it has been recently demonstrated that BRCA1/2 mutations in patients and BRCA1 silencing in cancer cell lines correlated with telomeric structural alterations such as hypomethylation of subtelomeric regions (in particular on chromosome X), increase of DDR at telomeres, and changes in telomere length [33]. To determine the molecular effects of BRCA2 loss on telomere homeostasis, we analyzed the isogenic system of colon cancer cell line DLD1 bearing homozygous knockout for BRCA2 (Figure 1A) [28]. Here we found a huge increase in total TERRA expression (Figure 1B) that correlated with a strong upregulation of transcription of subtelomeres 10q1 and 10q2, and with minor extent subtelomeres xyq1 and xyq2, which are recognized as the most active TERRA promoters [32]. TERRA hypertranscription was also confirmed in a HCT116 colon cancer cell line stably transfected with shRNA against BRCA2 or scrambled sequence (Supplemental Figure S1). 

### 3.2. BRCA2 Depletion Stimulates ALT Activity 

**Figure 2:**
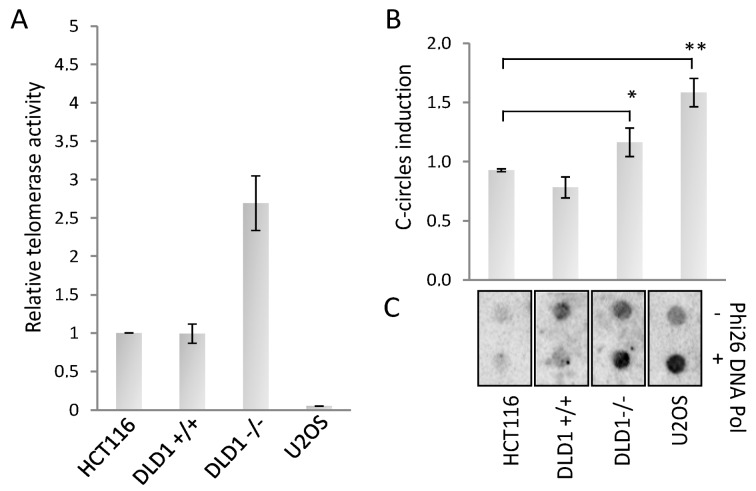
(**A**): Real-time quantitative telomerase repeat amplification protocol (TRAP) assay and c-circle assay. (**A**): Real-time qTRAP to determine the telomerase activity in the indicated cell lines. Histogram represents the fold change of telomerase activity compared to HCT116 sample. (**B**): Dot blot analysis of c-circles in presence or absence of Phi26 DNA Pol enzyme in the indicated cell lines, hybridized with a 32P radiolabeled telo-probe. (**C**): Densitometry of c-circles signals. For each cell line, the background value (-Phi26 DNA Pol sample) was subtracted and reported in histograms. The mean of three independent experiments with comparable results is shown. Bars are SD. * *p* < 0.05; ** *p* < 0.01.

The increase of TERRA expression is a characteristic of ALT cancers, and it was proposed that TERRA association with ALT telomeres could trigger recombination and elongation. To understand if BRCA2 could trigger a switch from telomerase to ALT, as first we measured the telomerase activity in both cell lines in comparison with HCT116 and U2OS (as telomerase positive and negative controls, respectively). We found that DLD1 proficient cells had a telomerase activity comparable with HCT116, surprisingly BRCA2 depletion showed an increase of telomerase activity, in spite of a reduction of hTERT mRNA expression level (Figure 2A and Supplemental Figure S2). Since telomerase activity and ALT can coexist in cancer cells, we measured the presence of ALT activity by the c-circle assay, a robust and quantitative assay to determine the presence of recombination at telomeres [34]. BRCA2 deletion strongly increased c-circle signals compared to proficient cell line and to HCT116 negative control, U2OS cell line is shown as a control of ALT positivity (Figure 2B). These results clearly indicate the presence of ALT activity in BRCA deficient cells. 

### 3.3. BRCA Deficient Cells Display Heterogeneous Telomere Length and APBs Induction

**Figure 3:**
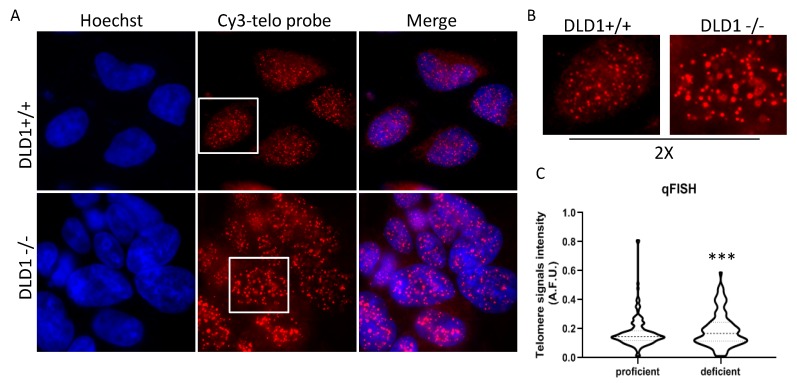
Q-FISH analysis of telomeric signals in BRCA2 proficient and deficient DLD1 cells. (**A**): Representative images acquired at 100× magnification. (**B**): 2× enlargements. (**C**): Violin plots showing the quantitative analysis of telomeric spots measured by ImageJ (60 nuclei per sample). F test, *p*-value: 0.0006 (*** *p* < 0.005).

HR between sister telomeres is a casual event that generates very heterogeneous telomeres, depending on the recombination site. Then, ALT cells are characterized by a higher average telomere length, but also by the presence of extremely long and short telomeres. To understand if BRCA2 depletion led to any significant change in telomere length, a qFISH analysis in interphase nuclei of both populations was performed by using a fluorescent telomeric probe, and the signals were quantified. As shown in Figure 3A, in nuclei of proficient cells, telomeric signals were more numerous compared to deficient ones and of homogeneous intensity. Contrarily, in deficient cells, the number of telomeric spots was lower and very high-intensity spots were present. This demonstrates an increase in heterogeneity of telomere length with the generation of very short (undetectable) and very long telomeres. The analysis of frequency variation showed a significantly different distribution of signal intensities between the two population (Figure 3B). 

**Figure 4:**
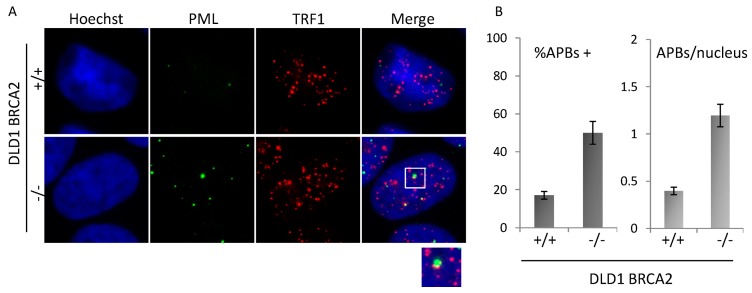
APBs analysis. (**A**): Representative images acquired at 100× magnification of BRCA2 proficient and deficient DLD1 co-immunolabeled for PML and TRF1. Representative image of co-localizations (APBs) is shown in the enlargement (2×). (**B**): Quantitative analysis of APBs in the two isogenic populations. Histograms represent the percentage of cells displaying at least 1 APB and the average number of APBs per nucleus. Images are representative of three independent experiments. Bars are SD.

Finally, the presence of ALT-associated PML-bodies (APBs) was scored in the two populations by co-immunofluorescence staining with anti-PML and TRF1 antibody. Co-localizing spots, defined as APBs, were analyzed by immunofluorescence microscopy (Figure 4A) and scored on more than 200 nuclei in triplicate samples each line. Data plotted in the histograms (Figure 4B) show induction of both the percentage of cells displaying at least 1 APB and of the mean number of APBs per nucleus.

## 4. Discussion

Telomere homeostasis is a prerequisite for cancer development and requires the presence of TMM whose mechanisms of activation are still not completely elucidated. BRCA2 protein exerts its main function in the repair of DSBs by loading RAD51 on the ssDNA and favoring double-strand invasion and HR. For this reason, BRCA2 deficiency confers synthetic lethality to the inhibition of other HR factors like PARP1. Our data, in line with previous evidence [33], indicate that BRCA2 loss could enhance ALT frequency, which cannot depend from BRCA2/RAD51 pathway that is abrogated in the DLD1 knockout system employed [28]. This, in agreement with previous evidence demonstrating demethylation of subtelomeric regions [33], is accompanied by a huge increase of TERRA transcription. The fact that BRCA2 acts as a suppressor of ALT is in apparent contradiction with the essential role of BRCA2 in HR required for ALT activity. Anyway, HR in ALT is not always dependent on the BRCA2/RAD51 axis. Instead, BRCA2 depletion, and the consequent RAD51 loss of function, was shown to direct HR pathway toward a Mre11 and RAD52 dependent break-induced replication (BIR) [35]. Data presented here also demonstrated that the BRCA2 depletion induced ALT activity in a telomerase positive background, although in these cell lines both telomerase activity and hTERT expression seem to be somehow affected, with unknown mechanisms that we will be interesting to better investigate in the future. However, this observation can account for the fact that ALT activation is not an escape mechanism of a surviving clone, but it coexists with telomerase activity. This implies that BRCA2 mutated (or BRCAness) cancers could not be suitable for anti-telomerase therapies, since they can intrinsically possess ALT activity that rescue proliferative potential of cancer cells. In addition, telomeric chromatin possesses several structural and epigenetic phenotypes. As first, the presence of GC-rich repeats allows telomeric single-strand loops to fold into G-quadruplex structures that may originate from the lagging strand of a replication fork or by r-loops formed by TERRA-DNA hybrids which are in fact more abundant in ALT cells [36,37]. In consideration of this, our data support a view in which ALT mechanism could be at the basis of a higher sensitivity of BRCA2 cells to some G-quadruplex ligands such as Pyridostatin and CX-5461 [29,38]. 

## Supplementary Materials

The following are available online at https://www.mdpi.com/2073-4425/10/9/697/s1, Figure S1: TERRA expression in HCT BRCA KD cells, Figure S2: hTERT expression in DLD1 BRCA KO cells.
