# Supplementary material for: BRCA2 Deletion Induces Alternative Lengthening of Telomeres in Telomerase Positive Colon Cancer Cells
Source: Genes (Basel). 2019 Sep 10;10(9):697. doi: 10.3390/genes10090697 (PMC6771010; doi:10.3390/genes10090697)
Supplement: Supplementary file 1 [file genes-10-00697-s001.pdf]

Supplementary Figure 1

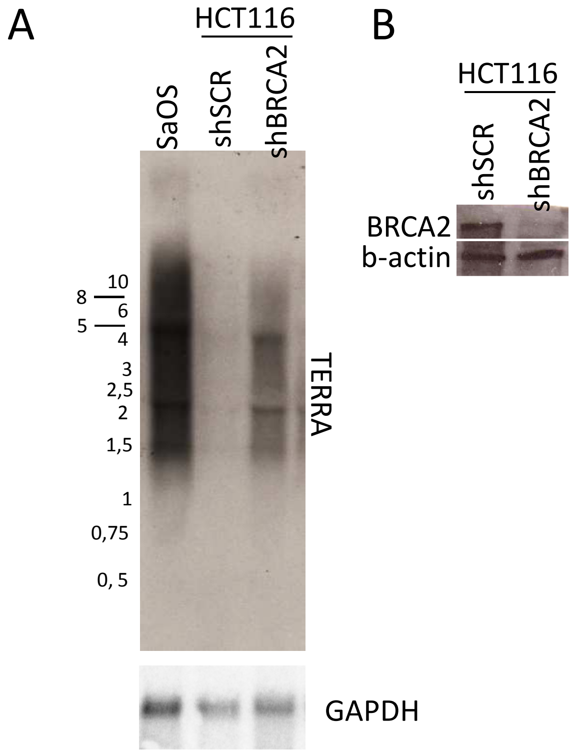

Supplementary Figure 1. A: TERRA expression in BRCA2 interfered HCT116 cells. Northern blot analysis of TERRA expression in the indicated cell lines, GAPDH is shown as loading control. B:western blot analysis of BRCA2 expression levels in HCT116 stably interfered for BRCA2 or scrambled sequence. b-actin was shown as loading control. One representative of three independent experiments is shown.

Supplementary Figure 2

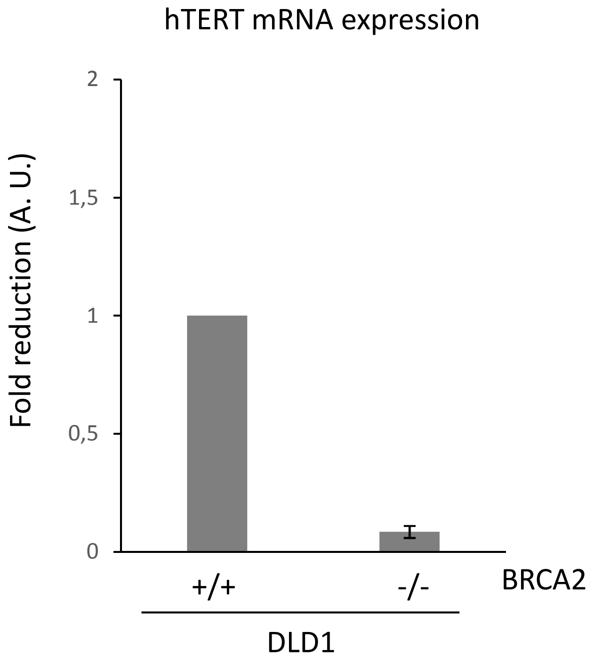

Supplementary Figure 2. hTERT mRNA expression levels upon BRCA2 depletion. BRCA2 deficient and proficient cells were grown at 50% confluence and then recovered. RNA was extracted with Trizol reagent (Invitrogen), and converted to cDNA with the SuperScript ®VILO™kit (Invitrogen). Real-time PCR was performed in triplicate using the 7900HT Fast Real Time PCR System (Applied Biosystem, Waltham, MA, USA). The following primers were used: fw: 5'TGT TTC TGG ATT TGC AGG TG-3'; rev: 5'GTT CTT GGC TTT CAG GAT GG-3'. The specificity of each PCR products was controlled using the melting curve. The relative gene expression levels were calculated using the 2-ΔΔCt method, where Ct represents the threshold cycle, and beta-actin was used as a reference gene. Histograms represent the fold reduction of BRCA2 mRNA level in DLD1-/- vs +/+ cell line. The mean of three independent experiments with comparable results is shown. Bars are SD.
